# Association between critical limb ischemia and arterial stiffness measured by brachial artery oscillometry

**DOI:** 10.1590/1677-5449.007318

**Published:** 2019-03-28

**Authors:** Daniel Mendes-Pinto, José Márcio Ribeiro, Maria da Glória Rodrigues-Machado

**Affiliations:** 1 Hospital Felício Rocho, Departamento de Cirurgia Vascular, Belo Horizonte, MG, Brasil.; 2 Faculdade de Ciências Médicas de Minas Gerais – FCM-MG, Belo Horizonte, MG, Brasil.; 3 Hospital Felício Rocho, Departamento de Cardiologia, Belo Horizonte, MG, Brasil.

**Keywords:** arterial stiffness, peripheral arterial disease, pulse wave analysis, ankle brachial index, rigidez arterial, doença arterial periférica, análise da onda de pulso, índice tornozelo-braço

## Abstract

**Background:**

Elevated arterial stiffness is associated with increased cardiovascular mortality. The relationship between arterial stiffness and critical limb ischemia (CLI) is not well established.

**Objectives:**

The objective of this study is to analyze the relationship between arterial stiffness indices and the degree of limb ischemia measured by the ankle-brachial index (ABI).

**Methods:**

A cross-sectional study comparing patients with CLI and controls. Arterial stiffness was measured using brachial artery oscillometry. The arterial stiffness indices pulse wave velocity (PWV) and augmentation index normalized to 75 beats/min (AIx@75) were determined. Multiple linear regression was applied to identify predictors of arterial stiffness indices.

**Results:**

Patients in the CLI group had higher PWV (12.1±1.9 m/s vs. 10.1±1.9 m/s, p < 0.01) and AIx@75 (31.8±7.8% vs. 17.5±10.8%, p < 0.01) than controls. Central systolic pressure was higher in the CLI group (129.2±18.4 mmHg vs. 115.2±13.1 mmHg, p < 0.01). There was an inverse relationship between AIx@75 and ABI (Pearson coefficient = 0.24, p = 0.048), but there was no relationship between ABI and PWV (Pearson coefficient = 0.19, p = 0.12). In multiple regression analysis, reduced ABI was a predictor of elevated levels of AIx@75 (β = -25.02, p < 0.01).

**Conclusions:**

Patients with CLI have high arterial stiffness measured by brachial artery oscillometry. The degree of limb ischemia, as measured by the ABI, is a predictor of increased AIx@75. The increased AIx@75 observed in CLI may have implications for the prognosis of this group of patients with advanced atherosclerosis.

## Resumo

**Contexto:**

A rigidez arterial aumentada está associada ao aumento da mortalidade cardiovascular. A relação entre rigidez arterial e isquemia crítica do membro (IC) não está bem estabelecida.

**Objetivos:**

O objetivo deste estudo é analisar a relação entre índices de rigidez arterial e o grau de isquemia de membro medido pelo índice tornozelo-braço (ITB).

**Métodos:**

Foi feito um estudo transversal em pacientes com IC e controles. A rigidez arterial foi medida usando a oscilometria da artéria braquial. Os índices de rigidez arterial mensurados foram a velocidade de onda de pulso (VOP) e o índice de aumentação corrigido para a frequência cardíaca de 75 batimentos/min (AIx@75). Regressão linear múltipla foi aplicada para identificar preditores dos índices de rigidez arterial.

**Resultados:**

Pacientes do grupo IC tiveram VOP (12,1±1,9 m/s vs. 10,1±1,9 m/s, p < 0,01) e AIx@75 (31,8±7,8% vs. 17,5±10,8%, p < 0,01) maiores que controles. Pressão sistólica central foi maior no grupo IC (129,2±18,4 mmHg vs. 115,2±13,1 mmHg, p < 0,01). Houve uma relação inversa entre o AIx@75 e o ITB (coeficiente de Pearson = 0,19, p = 0,12). A análise de regressão múltipla mostrou que o ITB reduzido foi um preditor de elevação do AIx@75 (β = -25,02, p < 0,01).

**Conclusões:**

Pacientes com IC têm elevada rigidez arterial medida por oscilometria da artéria braquial. O grau de isquemia do membro, medido pelo ITB, é um preditor do AIx@75 elevado. O aumento do AIx@75 na IC pode ter implicações de prognóstico no grupo de pacientes com aterosclerose avançada.

## INTRODUCTION

 Lower limb peripheral artery disease (PAD) is common in the elderly and its worldwide incidence is increasing. 1 Prevalence ranges from 18 to 20% in people over the age of 55 years and is slightly higher in women. Critical limb ischemia (CLI), the advanced stage of PAD, occurs in approximately 1.2% of the elderly population and the rate can be as high as 3.3% in groups older than 80 years. 2

 An ankle/brachial index (ABI) ≤ 0.90 is considered abnormal and when associated with ischemic pain at rest or ischemic wounds it confirms a diagnosis of CLI. 3 ^,^ 4 The ABI quantifies limb ischemia and is a marker of cardiovascular mortality. Identification of markers of progression of atherosclerosis in patients with CLI is important for classifying risk and defining the most appropriate treatment. 5

 Arterial stiffness reduces the elasticity of the arterial system and its ability to adjust to volume changes during the cardiac cycle. 6 Arterial stiffness has been analyzed in several groups of patients with the aim of classifying risk of cardiovascular mortality and establishing preventive measures or specific therapies. 7 The main indicator of arterial stiffness is pulse wave velocity (PWV), which is a measure of the speed at which the pulse wave travels in the arterial system. 7 Pulse wave velocity increases in atherosclerotic arteries with rigid walls. Thus, the volume entering the proximal aorta is ejected into a less compliant artery, which increases the pressure on the wall and causes ventricular overload. The augmentation index (AIx) and the same index corrected for a heart rate of 75 beats per minute (AIx@75) are indirect parameters of arterial stiffness that provide information about the pattern of pulse wave reflection after systolic ejection. 8 The AIx is an independent predictor of cardiovascular risk. 7 ^,^ 8

 Arterial stiffness measured by PWV and AIx has been analyzed in patients with PAD to establish criteria for determination of risk and prognosis. 9 Almost all of these studies were performed in patients with early stage, asymptomatic PAD or claudication. 10 ^-^ 13 There is no consensus on whether patients with advanced PAD have increased arterial stiffness. Brand et al. demonstrated that patients with CLI had high central aortic pulse pressures and AIx values, but a much reduced PWV. 14 It is important to determine risk of cardiovascular events in patients with CLI, because they are frequently submitted to interventions such as revascularizations and amputations. The objectives of this study were to evaluate whether patients with CLI had arterial stiffness indices greater than controls and to identify predictors of these indices. 

## METHODS

### Study population

 We evaluated patients seen at the Angiology and Vascular Surgery Department of a tertiary level hospital from May 2016 to April 2017. Patients aged 50 to 90 years were included in the study. Patients with no symptoms of PAD and with ABI between 0.91 and 1.30 were included in the control group. Those who presented with CLI characterized by pain at rest or by the presence of ischemic wounds and ABI ≤ 0.90 were included in the CLI group. 4 Patients with stable, asymptomatic arterial disease, Rutherford category 0 to 3, claudication, or ABI > 1.30 were excluded. Patients with acute arterial occlusion, acute myocardial infarction in the previous month, unstable angina, cerebrovascular accident, New York Heart Association class IV heart failure, or diagnosed malignancy were also excluded. The research project was approved by the Ethics and Research Committee under registration number 55440616.1.0000.5125. 

 The sample size was calculated to enable comparison of AIx and PWV between groups using independent samples. Given a significance level of 5% and a medium effect size, the number of individuals required to achieve a power of 90% was 70 in each group. The medium effect size was selected based on a study by Catalano et al., who found a 0.55 standard deviation difference in AIx between groups. 11

 Patients were examined by the lead author and by trained research assistants. Epidemiological data, biochemical parameters, measurements of arterial stiffness, and ABI were recorded. Smokers were defined as those who smoked at the time of the interview or who had stopped smoking at least 6 months prior to the study. Diabetes was defined as fasting glucose ≥ 126 mg/dL or use of insulin or oral hypoglycemic drugs. Hypertension was defined as systolic blood pressure ≥ 140 mmHg or diastolic blood pressure ≥ 90 mmHg during the interview or use of antihypertensive drugs. Dyslipidemia was defined as serum cholesterol above 200 mg/dL or use of a statin. History of coronary intervention or acute myocardial infarction (heart disease), cerebrovascular accident or transient ischemic attack, and congestive heart failure were all considered in the analysis. 

### Measurement of arterial stiffness and blood pressure

 Blood pressure (BP) at brachial level was measured in both arms with an aneroid sphygmomanometer and stethoscope, with the patient sitting and the limb resting on the examination table. Two pressure measurements were taken for each arm and the mean was considered for analysis. 

 Arterial stiffness indices were measured with a Mobil-O-Graph device (IEM; Stolberg, Germany). Brachial artery pulse wave capture was performed with a high-fidelity sensor (MPX5050; Freescale, Tempe, AZ, USA) incorporated into the cuff. After oscillometric measurement of BP, the cuff reinflates to diastolic BP level for 10 s and records information from the pulse waves. The aortic pulse wave is generated using a mathematical transfer function. 15 The software separates the waves by decomposing the aortic pulse wave into the ejected and reflected wave. 

 Several parameters were calculated. The AIx@75 was assessed from the central aortic pressure wave, which is characterized by two pressure peaks. The first peak is caused by contraction of the left ventricle and the second is the result of the reflection wave. The difference between the peaks represents the augmentation pressure (AP), which reflects the degree to which systolic blood pressure increases due to the reflection wave. AIx@75 is defined as the ratio of AP to central pulse pressure (PPc, the difference between systolic and diastolic pressure). AIx@75 reflects the increase in systolic pressure due to early return of reflected waves. The PWV is estimated using mathematical models that consider the parameters obtained by analysis of the pulse waves. The arterial stiffness indices PWV and AIX@75 were evaluated. The formula for AIx@75 is AIX@75 = AIx - 0.39 × (75 – heart rate). 16 Three consecutive measurements were made with the device positioned over the brachial artery in the dominant arm or in the arm with the higher systolic BP, with the patient seated. The mean of these three measurements was considered for analysis. 

 Data were obtained for stiffness indices, peripheral systolic blood pressure (SBPp), peripheral diastolic blood pressure (DBPp), peripheral pulse pressure (PPp), and mean arterial pressure (MAP), defined as MAP = (2DBPp+SBPp)/3. Central pressures and hemodynamic data, including central systolic blood pressure (SBPc), central diastolic blood pressure (DBPc), central pulse pressure (PPc), systolic volume, cardiac output, total vascular resistance (TVR), and the cardiac index (CI) were obtained. Medications in use by the patients were not suspended for data measurement. 

### Measurement of the ankle/brachial index

 The ABI was measured with the patient in the supine position, using portable continuous wave Doppler ultrasound with a 7.5-mHz probe (Microem, Ribeirão Preto, Brazil). Systolic pressures were measured bilaterally in the dorsalis pedis and posterior tibial arteries using Doppler ultrasound and an aneroid sphygmomanometer. The ABI of each lower limb was calculated by dividing the highest pressure in the tibial arteries by the highest systolic brachial pressure measured with the same sphygmomanometer. An ABI ≤ 0.90 was considered abnormal. 3 In patients without PAD, the lowest ABI was considered for analysis. 

### Statistical analysis

 Categorical variables were expressed as counts and percentages and quantitative variables as means ± standard deviation. Quantitative variables were compared using Student's *t* test or the Mann-Whitney test, as appropriate. Categorical variables were compared using the chi-square test and Fisher's exact test. Linear regression models were used to evaluate the effect of variables on AIx@75 and PWV. Variables with p<0.20 in the univariate analysis were included in a saturated model and assessed with a stepwise strategy. The quality of fit was assessed by the R^2^ value and residual analysis. Analyses were performed using the R program (The R Foundation, Auckland, New Zealand), version 3.3.2, and a significance level of 5% was adopted. 

## RESULTS

 A total of 312 patients were evaluated at first visit between May 2016 and April 2018. One hundred and two patients did not meet the inclusion criteria and 2 refused to participate. Complete hemodynamic analysis and arterial stiffness measurements were conducted for 142 patients: 70 in the control group and 72 in the CLI group. Two patients in the CLI group had pain at rest (Rutherford category 4), 68 had ischemic ulcers and were classified as Rutherford category 5, and 2 patients were considered category 6. 

 Patients in the control group were significantly taller than those in the CLI group (p = 0.01) ( Table 1 ). Although the proportion of diabetics was similar, there was a higher frequency of insulin users in the CLI group. Patients in this group also used antiplatelet drugs more frequently. 

**Table 1:** Clinical characteristics of the individuals in the Control and CLI groups.

**Variable**	**Whole sample** **(n = 142)**	**Control** **(n=70)**	**CLI** **(n=72)**	**p-value**
Age	73.4±10.2	71.5±10.2	74.1±9.7	0.11 M
Male sex	82 (57.7%)	41 (58.6%)	41 (56.9%)	1.00 Q
Weight	76.8±13.6	78.6±17.2	75.3±12.6	0.09 T
Height	1.69±0.18	1.71±0.10	1.66±0.11	0.01 M
Smokers	12 (8.4%)	5 (7.1%)	7 (9.7%)	0.59 F
Hypertension	102 (71.8%)	49 (70.0%)	53 (73.6%)	0.63^Q^
Diabetes	89 (67.4%)	41 (62.1%)	48 (72.7%)	0.32^Q^
Dyslipidemia	68 (51.9%)	29 (43.9%)	39 (60%)	0.13^Q^
Coronary disease	24 (18.2%)	6 (9.1%)	18 (27.3%)	< 0.01^Q^
Stroke	7 (5.3%)	2 (3.0%)	5 (7.6%)	0.29^F^
Antihypertensives	103 (78%)	51 (77.3%)	52 (78.8%)	0.93^Q^
Antidiabetics	81 (61.4%)	33 (50%)	48 (72.7%)	0.02^Q^
Insulin	43 (32.6%)	9 (13.6%)	34 (51.5%)	< 0.01^Q^
Statins	83 (62.9%)	39 (59.1%)	44 (66.7%)	0.52^Q^
Antiplatelets	66 (50%)	17 (25.8%)	49 (74.2%)	< 0.01^Q^
Anticoagulants	13 (9.8%)	9 (13.6%)	4 (6.1%)	0.14^F^

Data are expressed as mean ± standard deviation or absolute and relative frequencies. The p-values refer to the following tests:

M Mann-Whitney;

T Student’s *t* test for independent samples;

Q chi-square; and

F Fischer’s exact test.

**Figure 1:**
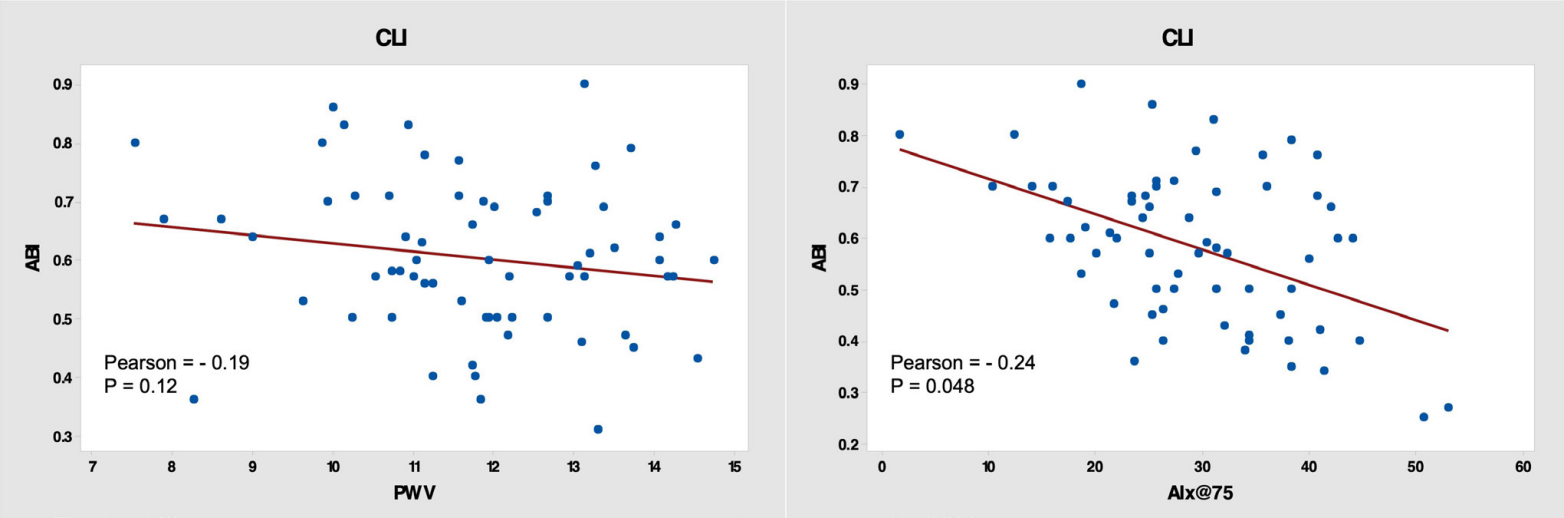
Linear correlation between ABI vs. PWV and ABI vs. AIx@75 in CLI patients. ABI = ankle/brachial index; PWV = pulse wave velocity; AIx@75 = augmentation index normalized to 75 beats/min; CLI = critical limb ischemia group.

 CLI patients had higher peripheral and central arterial blood pressure and TVR than those in the control group ( Table 2 ). Ischemic patients had higher arterial stiffness indices, AIx@75 (31.8 ± 7.8% vs. 17.5 ± 10.8%) and higher PWV (12.1 ± 1.9 m/s vs. 10.1 ± 1.9 m/s). There was no correlation between ABI and PWV (Pearson correlation coefficient = 0.19, p=0.12). There was a weak inverse correlation between ABI and AIx@75 (Pearson correlation coefficient = 0.24, p=0.048) ( Figure 1 ). 

**Table 2:** Parameters of peripheral arterial pressure, central pressure, hemodynamics, arterial stiffness indices and ankle/brachial index of the study population.

**Variable**	**All** **(n = 142)**	**Control** **(n = 70)**	**CLI** **(n = 72)**	**p-value**
Peripheral arterial pressure				
SBPp (mmHg)	139.6±20.1	122.2±11.7	146.8±21.7	< 0.01 M
DBPp (mmHg)	81.5±10.9	77.1±8.9	84.5±11.9	0.02 T
MAP (mmHg)	103.5±13.2	97.5±10.6	112.2±15.3	< 0.01^M^
HR (bpm/min)	72.9±11.8	72.7±11.4	72.1±13.2	0.97^M^
Central arterial pressure				
SBPc (mmHg)	121.7±15.8	115.2±13.1	129.2±18.4	< 0.01^M^
DBPc (mmHg)	83.2±10.2	78.2±8.1	86.1±11.2	< 0.01^T^
PPc (mmHg)	42.2±11.4	38.4±7.2	46.6±14.3	< 0.01^M^
Hemodynamics				
Systolic volume (ml)	72.0±10.8	73.2±15.1	72.1±18.1	0.67^M^
Cardiac output (l/min)	5.3±0.4	5.2±0.6	5.2±1.3	0.84^M^
TVR (s*mmHg/ml)	1.8±0.4	1.8±0.3	2.1±0.3	< 0.01^M^
Cardiac index (l/min*1/m^2^)	2.9±0.6	2.9±0.4	2.9±0.5	0.91^M^
Arterial stiffness				
AIx@75 (%)	25.2±11.2	17.5±10.8	31.8±7.8	< 0.01^T^
PWV (m/s)	11.0±1.2	10.1±1.9	12.1±1.9	< 0.01^T^
ABI	0.79±0.8	1.03±0.1	0.54±0.2	< 0.01^M^

Data are expressed as mean ± standard deviation or absolute and relative frequencies. The p-values refer to the following tests:

M Mann-Whitney;

T Student’s t test for independent samples;

ABI = ankle/brachial index; AIx@75 = augmentation index normalized to 75 beats/min; DBPc = central diastolic blood pressure; DBPp = peripheral diastolic blood pressure; HR = heart rate; MAP = mean arterial pressure; PPc = central pulse pressure; PWV = pulse wave velocity; SBPc = central systolic blood pressure; SBPp = peripheral systolic blood pressure; TVR = total vascular resistance; CLI = critical limb ischemia group.

 The variables that influenced AIx@75 after multiple linear regression analysis are shown in Table 3 , together with the univariate analysis of these data. In the control group, male sex (β = -9.22, p < 0.01) and insulin use (β = -6.45, p < 0.01) were inverse predictors and correlated with reduced levels of AIx@75. In this group, age was a predictor of elevated AIx@75 levels (β = 0.43, p > 0.12). In the CLI group, diabetes was a positive predictor, correlated with elevated AIx@75 levels (β= 8.86, p < 0.01). In this group, there was an inverse correlation between ABI and AIx@75 (β = -25.02, p <0.01), so low ABI levels were predictive of elevated AIx@75 levels. 

**Table 3:** Univariate regression analysis and multiple linear regression model using AIx@75 as the dependent variable.

**Variable**	**Univariate regression analysis**				**Multiple linear regression analysis**			
	**CONTROL**		**CLI**		**CONTROL**		**CLI**	
	**β (SE)**	**p-value**	**β (SE)**	**p-value**	**β (SE)**	**p-value**	**β (SE)**	**p-value**
Male sex	-7.99 (2.11)	0.01	-6.02 (2.18)	0.01	-9.22 (1.85)	< 0.01	-	-
Age	-0.03 (0.12)	0.77	0.28 (0.12)	0.03	0.43 (0.12)	< 0.01	-	-
Diabetes	-3.32 (2.55)	0.21	7.94 (2.23)	< 0.01	-	-	8.86 (1.58)	< 0.01
Insulin use	-9.97 (3.52)	0.07	2.03 (2.57)	0.57	-6.45 (3.19)	< 0.01	-	-
ABI	-16.19 (12.54)	0.20	- 19.29 (9.48)	0.04	-	-	-25.02 (9.75)	< 0.01

ABI = ankle/brachial index; AIx@75 = augmentation index normalized to 75 beats/min; CLI = critical limb ischemia group; SE = standard error. Normal distribution of residuals, without outliers and without violation of homoscedasticity.

Table 4 shows the variables that influenced PWV after multiple linear regression analysis. In both control and CLI groups there were positive correlations between age and PWV and between SBPp and PWV. 

**Table 4:** Univariate regression analysis and multiple linear regression model using PWV as the dependent variable.

**Variable**	**Univariate regression analysis**				**Multiple linear regression analysis**			
	**CONTROL**		**CLI**		**CONTROL**		**CLI**	
	**β (SE)**	**p-value**	**β (SE)**	**p-value**	**β (SE)**	**p-value**	**β (SE)**	**p-value**
Age	0.12 (0.02)	< 0.01	0.13 (0.01)	< 0.01	0.16 (0.01)	< 0.01	0.17 (0.01)	< 0.01
SBPp	0.02 (0.01)	< 0.01	0.03 (0.01)	< 0.01	0.03 (0.01)	< 0.01	0.03 (0.01)	< 0.01

CLI = critical limb ischemia group; PWV = pulse wave velocity; SBPp = peripheral systolic blood pressure; SE = standard error. Normal distribution of residuals, without outliers and without violation of homoscedasticity.

## DISCUSSION

 This study showed that patients with CLI had higher values of arterial stiffness indices compared to controls, matched by gender and age. There was a correlation between the degree of limb ischemia as measured by ABI and arterial stiffness as measured by AIx@75. These data suggest that the more advanced the limb ischemia measured by ABI, the greater the magnitude of reflected waves measured by AIx@75. Central arterial pressures were also higher in the CLI group, which is consistent with advanced atherosclerotic disease. 17

 Arterial stiffness has been analyzed in groups of patients with advanced atherosclerotic disease, such as those with coronary disease or on hemodialysis, with the objective of risk classification and to determine prognosis of cardiovascular events. 18 For patients with PAD, arterial stiffness has mainly been analyzed in claudicants, also with the objective of assisting cardiovascular risk analysis. 10 ^-^ 13 There are few studies evaluating patients with an advanced stage of PAD. 14

 The main explanation for the association between low ABI levels and high AIx@75 levels is the fact that multisegmental arterial obstruction in the lower limbs can lead to early pulse wave reflections from the peripheral circulation to the aorta. Additionally, the reduction of the capillary bed in the distal circulation that occurs in advanced atherosclerosis increases pulse wave reflections. 8 The association between arterial stiffness and ABI, which is the main marker of PAD, has been studied by other authors. In a community sample of 475 patients, 59 had ABI < 1.0, and this subgroup had higher AIx values. 19 In a study of 4,159 subjects in the general population, higher AIx levels correlated inversely with ABI. 20

 In the current study, patients with limb ischemia had higher PWV than those in the control group. However, there was no relationship between PWV and the degree of ischemia measured by ABI. It’s important to consider that even the control group had an elevated PWV value, of 12.1±1.9 m/s. A PWV above 10 m/s has been associated with increased mortality. 21 The high PWV in the control group can be explained by the elevated age of these patients. 

 In the only study we found with CLI patients, Brand et al. reported different findings. 14 These authors identified a low PWV of 5.72 m/s in a sample of 136 patients with CLI. Despite the low PWV, the sample had high AIx and pulse pressure. The authors accounted for the reduced PWV by postulating that the reduction in femoral artery pulse amplitude captured by tonometry was due to obstructions in proximal arterial segments. Thus, the increase in transit time during pulse wave capture over the femoral artery resulted in a lower PWV value. The difference in methodologies used for PWV measurement explains the differences between the data observed in the present study and those reported by Brand et al. In our study, PWV was measured using brachial artery oscillometry, which is less affected by atherosclerotic obstruction than aortoiliac or infra-inguinal segments. 

 Our finding of an inverse correlation between male sex and the AIx@75 is consistent with published data. Populational studies show that women have higher PWV and AIx than men. 22 ^,^ 23 Anatomic factors such as the reduction in arterial diameter and decrease in microcirculation that occurs after menopause explain the progression of atherosclerosis in women of advancing age. 24

 Advanced age is associated with increased stiffness of the arterial wall. 11 ^,^ 22 In our sample, the influence of age on indicators of arterial stiffness was small, as shown by the regression beta coefficients. One possible explanation for the minor effect of age on arterial stiffness indices is the fact that the sample was composed of elderly patients, with little variation in age. 

 Diabetes has been linked to increased arterial stiffness for several reasons. There is a reduction of the distensibility of muscular arteries due to the progression of atherosclerosis, reduction of capillaries in target organs, and thickening and calcification of the tunica media of small and medium-sized arteries. 25 Our data show a positive correlation between the presence of diabetes and AIx@75, which is consistent with the literature. 26

 High levels of SBPp were associated with high PWV. Increased systolic pressure with maintenance of diastolic pressure is associated with an increase in pulse pressure, which is a determinant of PWV, in addition to being associated with cardiovascular complications. 27

 Measurement of pulse waves by brachial artery oscillometry, as used in this study, is a validated method when compared with other invasive and noninvasive methods of arterial stiffness measurement. 28 ^,^ 29 Correlation of stiffness indices measured by brachial artery oscillometry in patients with PAD is a new finding in the literature. This method has the advantage of being relatively simple and less operator-dependent than other methods used for arterial stiffness measurement. 15

 One limitation of this study is its cross-sectional character, which prevents determination of any temporal association between PAD and increased arterial stiffness. We chose to include patients with the ABI ≤ 0.90 in the CLI group because this is the most commonly used definition for patients with peripheral arterial disease. However, patients with critical ischemia are expected to have lower ABI values. Measurement of toe pressure would be more accurate for characterization of CLI, but this measurement was not performed, which was a limitation of the study. Additionally, the sample included patients who were referred due to circulatory problems and many were scheduled for surgery. Their medications were therefore not discontinued for evaluation of arterial stiffness. 

 In conclusion, this study shows that the degree of limb ischemia as measured by ABI is related to the intensity of pulse wave reflections as measured by AIx@75. This relationship helps to classify the risk of cardiovascular events in patients with PAD, since the increase in arterial stiffness is associated with several cardiovascular complications. Since this relationship has been established, defining the prognostic value of arterial stiffness indices in longitudinal studies may help to define therapeutic measures in the population with advanced PAD. 

## References

[1] Fowkes FGR, Rudan D, Rudan I (2013). Comparison of global estimates of prevalence and risk factors for peripheral artery disease in 2000 and 2010: a systematic review and analysis. Lancet.

[2] Criqui MH, Aboyans V (2015). Epidemiology of peripheral artery disease. Circ Res.

[3] Gerhard-Herman MD, Gornik HL, Barrett C (2017). 2016 AHA/ACC Guideline on the management of patients with lower extremity peripheral artery disease: executive summary: a report of the American College of Cardiology/American Heart Association Task Force on Clinical Practice Guidelines. Circulation.

[4] Aboyans V, Ricco JB, Bartelink MLEL (2018). Editor’s Choice - 2017 ESC guidelines on the diagnosis and treatment of peripheral arterial diseases, in collaboration with the European Society for Vascular Surgery (ESVS). Eur J Vasc Endovasc Surg.

[5] Vlachopoulos C, Xaplanteris P, Aboyans V (2015). The role of vascular biomarkers for primary and secondary prevention: a position paper from the European Society of Cardiology Working Group on peripheral circulation. Endorsed by the Association for Research into Arterial Structure and Physiology (ARTERY) Society. Atherosclerosis.

[6] Boutouyrie P, Briet M, Collin C, Vermeersch S, Pannier B (2009). Assessment of pulse wave velocity. Artery Res.

[7] Townsend RR, Wilkinson IB, Schiffrin EL (2015). Recommendations for improving and standardizing vascular research on arterial stiffness: a scientific statement from the American Heart Association. Hypertension.

[8] Chirinos JA, Kips JG, Jacobs DR (2012). Arterial wave reflections and incident cardiovascular events and heart failure: MESA (Multiethnic Study of Atherosclerosis). J Am Coll Cardiol.

[9] Kals J, Lieberg J, Kampus P, Zagura M, Eha J, Zilmer M (2014). Prognostic impact of arterial stiffness in patients with symptomatic peripheral arterial disease. Eur J Vasc Endovasc Surg.

[10] Zahner GJ, Gruendl MA, Spaulding KA (2017). Association between arterial stiffness and peripheral artery disease as measured by radial artery tonometry. J Vasc Surg.

[11] Catalano M, Scandale G, Carzaniga G (2014). Aortic augmentation index in patients with peripheral arterial disease. J Clin Hypertens.

[12] Jacomella V, Shenoy A, Mosimann K, Kohler MK, Amann-Vesti B, Husmann M (2013). The impact of endovascular lower-limb revascularisation on the aortic augmentation index in patients with peripheral arterial disease. Eur J Vasc Endovasc Surg.

[13] Zahner GJ, Spaulding KA, Ramirez JL (2018). Characterizing the relationship between flow-mediated vasodilation and radial artery tonometry in peripheral artery disease. J Surg Res.

[14] Brand M, Woodiwiss AJ, Michel F, Booysen HL, Veller MG, Norton GR (2013). A mismatch between aortic pulse pressure and pulse wave velocity predicts advanced peripheral arterial disease. Eur J Vasc Endovasc Surg.

[15] Weiss W, Gohlisch C, Harsch-Gladisch C, Tölle M, Zidek W, Van Der Giet M (2012). Oscillometric estimation of central blood pressure: Validation of the Mobil-O-Graph in comparison with the SphygmoCor device. Blood Press Monit.

[16] Gallagher D, Adji A, O’Rourke MF (2004). Validation of the transfer function technique for generating central from peripheral upper limb pressure waveform. Am J Hypertens.

[17] Beckmann M, Jacomella V, Kohler M (2015). Risk stratification of patients with peripheral arterial disease and abdominal aortic aneurysm using aortic augmentation index. PLoS One.

[18] Sutton-Tyrrell K, Najjar SS, Boudreau RM (2005). Elevated aortic pulse wave velocity, a marker of arterial stiffness, predicts cardiovascular events in well-functioning older adults. Circulation.

[19] Khaleghi M, Kullo IJ (2007). Aortic augmentation index is associated with the ankle-brachial index: a community-based study. Atherosclerosis.

[20] Eldrup N, Sillesen H, Prescott E, Nordestgaard BG (2006). Ankle brachial index, C-reactive protein, and central augmentation index to identify individuals with severe atherosclerosis. Eur Heart J.

[21] Mattace-Raso F, Hofman A, Verwoert GC (2010). Determinants of pulse wave velocity in healthy people and in the presence of cardiovascular risk factors: ‘establishing normal and reference values.’. Eur Heart J.

[22] Nunan D, Wassertheurer S, Lasserson D (2012). Assessment of central haemomodynamics from a brachial cuff in a community setting. BMC Cardiovasc Disord.

[23] Janner JH, Godtfredsen NS, Ladelund S, Vestbo J, Prescott E (2010). Aortic augmentation index: Reference values in a large unselected population by means of the sphygmocor device. Am J Hypertens.

[24] Teodorescu VJ, Vavra AK, Kibbe MR (2013). Peripheral arterial disease in women. J Vasc Surg.

[25] Yannoutsos A, Ahouah M, Tubiana CD (2016). Hemodynamic parameters in hypertensive diabetic patients. J Hypertens.

[26] Kozakova M, Palombo C (2016). Diabetes mellitus, arterial wall, and cardiovascular risk assessment. Int J Environ Res Public Health.

[27] Zettervall SL, Buck DB, Darling JD, Lee V, Schermerhorn ML, Guzman RJ (2016). Increased preoperative pulse pressure predicts procedural complications and mortality in patients who undergo tibial interventions for critical limb ischemia. J Vasc Surg.

[28] Kollias A, Protogerou AD, Stergiou GS (2017). Antihypertensive treatment-induced changes in arterial stiffness. J Hypertens.

[29] Hametner B, Wassertheurer S, Kropf J, Mayer C, Eber B, Weber T (2013). Oscillometric estimation of aortic pulse wave velocity: Comparison with intra-aortic catheter measurements. Blood Press Monit.

